# Performance of *Salvinia molesta* plants in tertiary treatment of domestic wastewater

**DOI:** 10.1016/j.heliyon.2021.e06040

**Published:** 2021-01-24

**Authors:** Hauwa Mohammed Mustafa, Gasim Hayder

**Affiliations:** a College of Graduate Studies, Universiti Tenaga Nasional (UNITEN), 43000 Kajang, Selangor Darul Ehsan, Malaysia; b Department of Chemistry, Kaduna State University (KASU), Tafawa Balewa Way, P.M.B. 2339, Kaduna, Nigeria; c Institute of Energy Infrastructure (IEI), Universiti Tenaga Nasional (UNITEN), 43000 Kajang, Selangor Darul Ehsan, Malaysia; d Department of Civil Engineering, College of Engineering, Universiti Tenaga Nasional (UNITEN), 43000 Kajang, Selangor Darul Ehsan, Malaysia

**Keywords:** pH, Chemical oxygen demand (COD), Colour, Biological oxygen demand (BOD_5_), Hydroponic tanks, Biological treatment method

## Abstract

The objective of this study was to investigate the performance of different weight of *Salvinia molesta* plants in biological treatment of domestic wastewater. Three treatment systems containing 280g (GS1), 140g (GS2) and 70g (GS3) of *S. molesta* plants were used for the phytoremediation process. Physicochemical analysis such as pH, colour, chemical oxygen demand (COD), and biological oxygen demand (BOD_5_) of the influent and effluent water samples were performed according to spectrophotometric methods. The outcome of the study demonstrated that the different weight of *S. molesta* plants played a significant role in improving the quality of the wastewater samples, in which GS1 removed 96.8% (colour), 91% (BOD_5_), and 82.6% (COD). While up to 88.6% (colour), 87.1% (BOD_5_), and 81.1% (COD) reduction was observed for GS2 treatment systems, and GS3 was efficient in removing 85.5% (colour), 86.1% (BOD_5_), and 68.3% (COD). Also, a pH value of 6.29–7.19, 5.97–7.07, and 6.17–7.42 was obtained from GS1, GS2 and GS3 treatment systems, respectively. Thus, the treatment system with the highest quantity of *S. molesta* (GS1) demonstrated better performance compared to the other two systems (GS2 and GS3). The findings of this research can be applied in addressing the goals of sustainable development through the use of green technology to reduce the threat of water pollution in natural water bodies. Perhaps existing and future water scarcity can be resolved through the use of phytoremediation technology.

## 1 Introduction

Pollution has been one of the core fundamental issues which affect our present-day society. High rise in human population, industrialization and urbanization over the last century has led to the generation and discharge of toxic waste materials into our environment [1]. Large water bodies in most countries have been contaminated by organic and inorganic compounds from domestic, agricultural, and industrial sewage [2]. In 2015, soil, water and waste were responsible for 16% of deaths worldwide with around 92% of these deaths recorded from developing countries [3]. Similarly, water pollution has negatively affected the agricultural sector leading to bioaccumulation of toxic metals such as heavy metals in the food chain [4].

However, despite the invention of several conventional water treatment methods involving biological and chemical procedures conducted in primary, secondary and tertiary stages, high cost of operation and maintenance, energy requirement, and generation of contaminated chemical sludge have hindered its application in industries and developing countries [5,6]. Hence, the development of an ecologically engineered biological treatment system that can complement the conventional treatment methods has become a priority for developing countries and industries [7]. Thus, phytoremediation techniques fit this description. Phytoremediation is a biological method of removing pollutants from wastewater, air, and soil using plants. As a result, aquatic plants such as floating, submerged and emergent plants have been applied in phytoremediation of wastewater. Ahmad et al. [8] evaluated the potentials of *Salvinia molesta, Azola pinnata* and *Scirpus grossus* in polishing of paper mill sewage. The outcome of the study indicated that the native tropical plants reduced the colour concentration of the wastewater samples up to 50.28%, 43.09% and 49.7% by *S. grossus*, *A. pinnata* and *S. molesta*, respectively. At the same time, 100% removal was observed for chemical oxygen demand (COD). Similarly, Kumar and Deswal [9] assessed the performance of *Eichhornia crassipes*, *Salvinia molesta*, *Lemna minor* and *Pistia stratiotes* in the reduction of phosphorous and COD from rice mill wastewater for 15 days. The result obtained indicated that up to 75% and 80% reduction were recorded for COD and phosphorus, respectively. Nevertheless, most of the existing literature on applications of *Salvinia molesta* plants focused on phytoremediation of industrial wastewater. Therefore, the objective of this study was to investigate the performance of different weight of *S. molesta* plants in improving the quality of treated domestic wastewater to allowable discharge limits. *Salvinia molesta* also known as giant salvinia is a free-floating fern from *Salviniaceae* family capable of absorbing toxic compounds from wastewater [6,10].

## 2 Materials and methods

### 2.1 Study site

**Figure 1:**
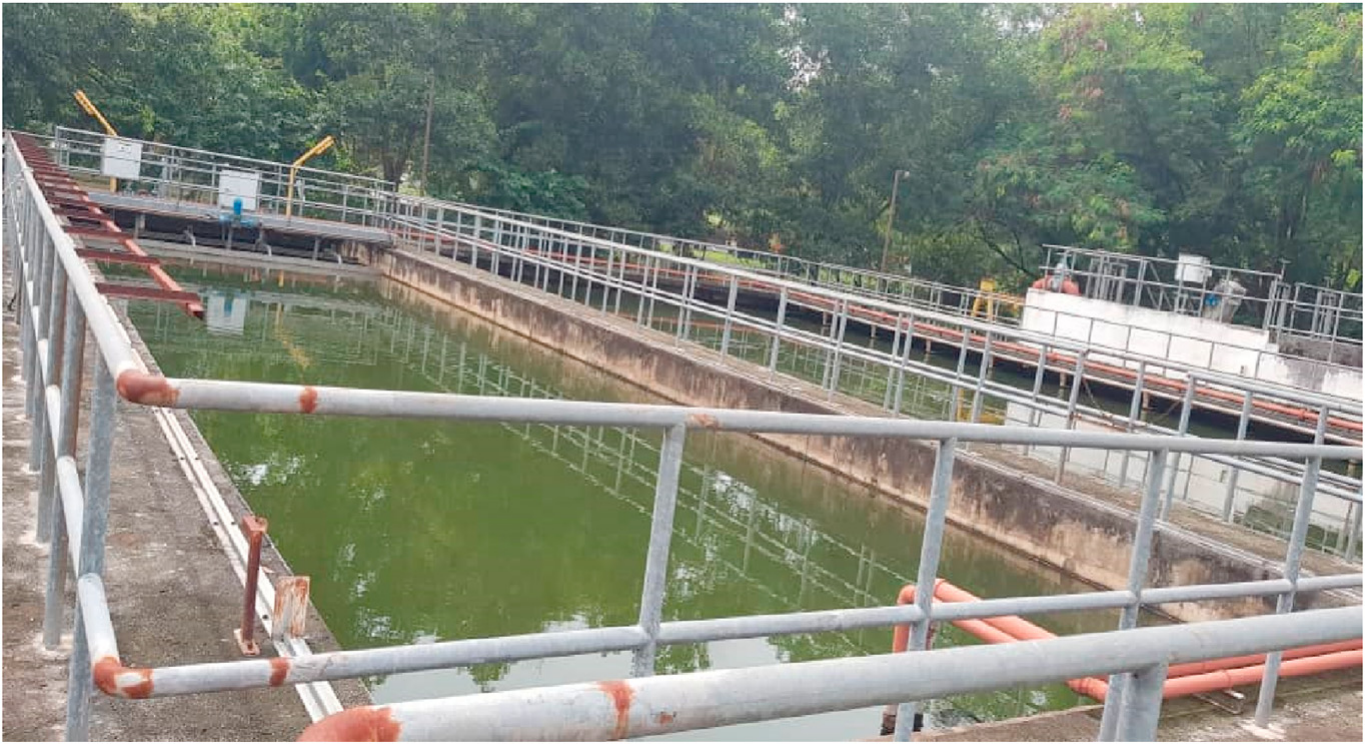
Section of the sewage treatment plant.

This research was performed at a Sewage Treatment Plant (STP). The STP is located within the perimeter of Kajang metropolis, Malaysia and it is saddled with the treatment of sewage that comes from the surrounding community. The sewage in the STP is treated through coagulation-flocculation methods before discharge into the environment. A section of the STP is presented in Figure 1.

### 2.2 Collection of the plant samples

**Figure 2:**
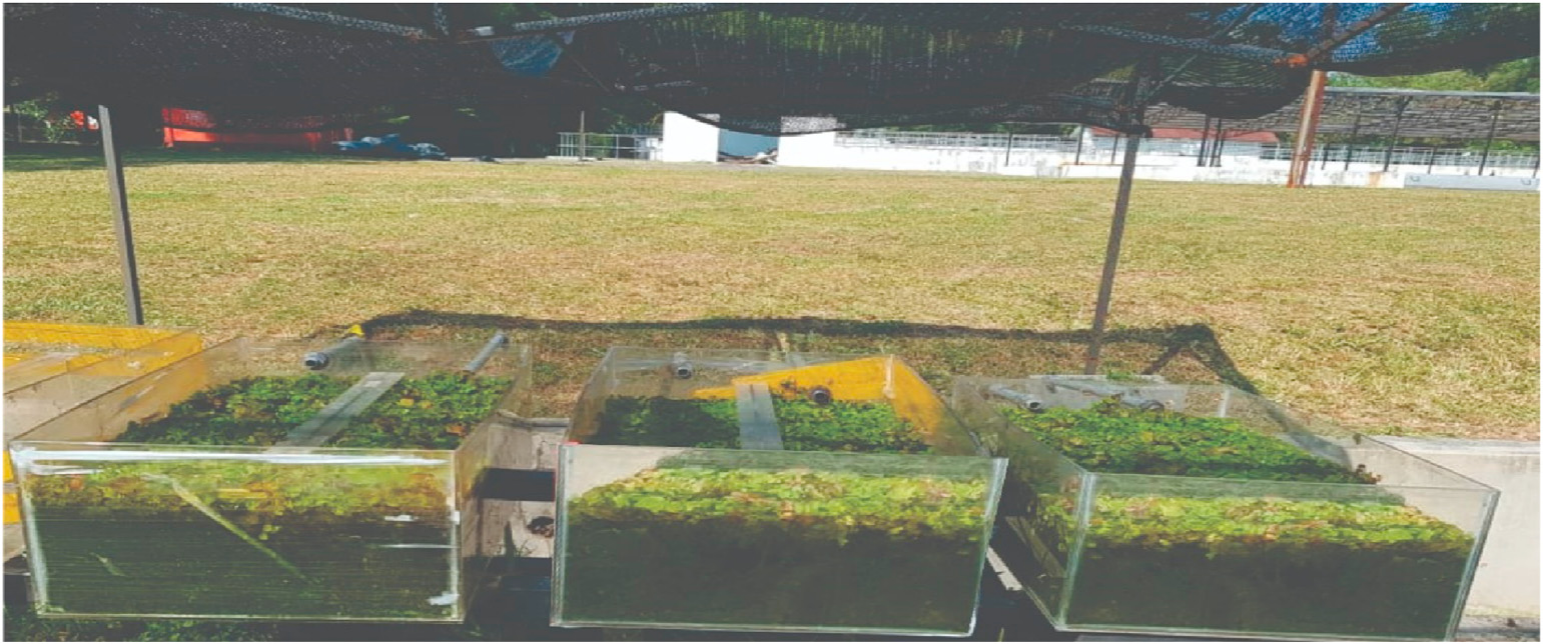
Cultivated *S. molesta* plant samples.

The *S. molesta* plants used in this research work was obtained from Kajang metropolis, Malaysia. The collected plant samples were washed with tap water to remove sand particles on the roots, before weighing 70g, 140g and 280g of the fresh plant samples into each of the three constructed hydroponic tanks. Besides, the plant weight was selected based on past research conducted by [11]. Furthermore, the plant samples were allowed to acclimatised for 5 days before the commencement of the sampling study. The diagram of the cultivated plant samples is presented in Figure 2.

### 2.3 Collection of domestic wastewater samples

This research was performed according to the methods described by [12,13]. The influent sample represents the secondary treated water from the exit point of the STP that is allowed to enter the treatment systems. While the effluent is the water samples collected during the cultivation of *S. molesta* plants at a retention time of 24 h for 14 days. The water samples were collected aseptically using sterile sampling bottles of 250ml volume. Effluent water samples collected from the treatment tank cultivated with 280g of *S. molesta* was labelled as GS1. At the same time, GS2 and GS3 represents effluent water samples collected from the treatment tank cultivated with 140g and 70g of *S. molesta* plants, respectively.

### 2.4 Physicochemical analysis

The influent and effluent water samples were subjected to physicochemical analysis such as pH, colour, chemical oxygen demand (COD), and biological oxygen demand (BOD_5_) using spectrophotometric methods described below.

#### 2.4.1 pH test of the water samples

The test for pH was performed by the use of pH meter (SCHEMLZ Rev V2.0, TPS Pty Ltd., Australia). The pH probe was continuously stirred in the water samples until a stable and accurate pH reading was obtained. Similarly, the pH analysis was carried out in the laboratory at a temperature of 25 ^O^C [14].

#### 2.4.2 Colour analysis of the wastewater samples

DR3900 spectrophotometer (HACH, Loveland, Co., USA), programmed on 120 colour 455nm was employed for the colour analysis of the influent and effluent water samples [15].

#### 2.4.3 Biochemical oxygen demand (BOD_5_) analysis of the water samples

BOD in water is usually calculated by the difference between the dissolved oxygen (DO) concentrations in the water samples before incubation and after 5 days of incubation [16]. DO meter with model HQ40d (HACH, Loveland, Co., USA) portable analyzer as described by the dilution method (method 8043) was used to measure the dissolved oxygen of the samples. The BOD_5_ was calculated using the mathematical formula (Eq. 1) [17]:

Eq. 1 $$BOD_{5}\left(mg/L\right)=\frac{DO_{initial}-DO_{final}}{volume\;of\;sample}$$

Where; $DO_{initial}$ is the initial dissolved oxygen and $DO_{final}$ is the final dissolved oxygen.

#### 2.4.4 Chemical oxygen demand analysis of the water samples

The COD analysis of the water samples was performed according to United States Environmental Protection Agency (USEPA) Reactor Digestion Method (8000) at 610 nm with DR 3900 spectrophotometer (HACH, Loveland, Co., USA) programmed on 430 COD LR detectable range of 3–150 mg/L [18].

### 2.5 Statistical analysis

**Figure 3:**
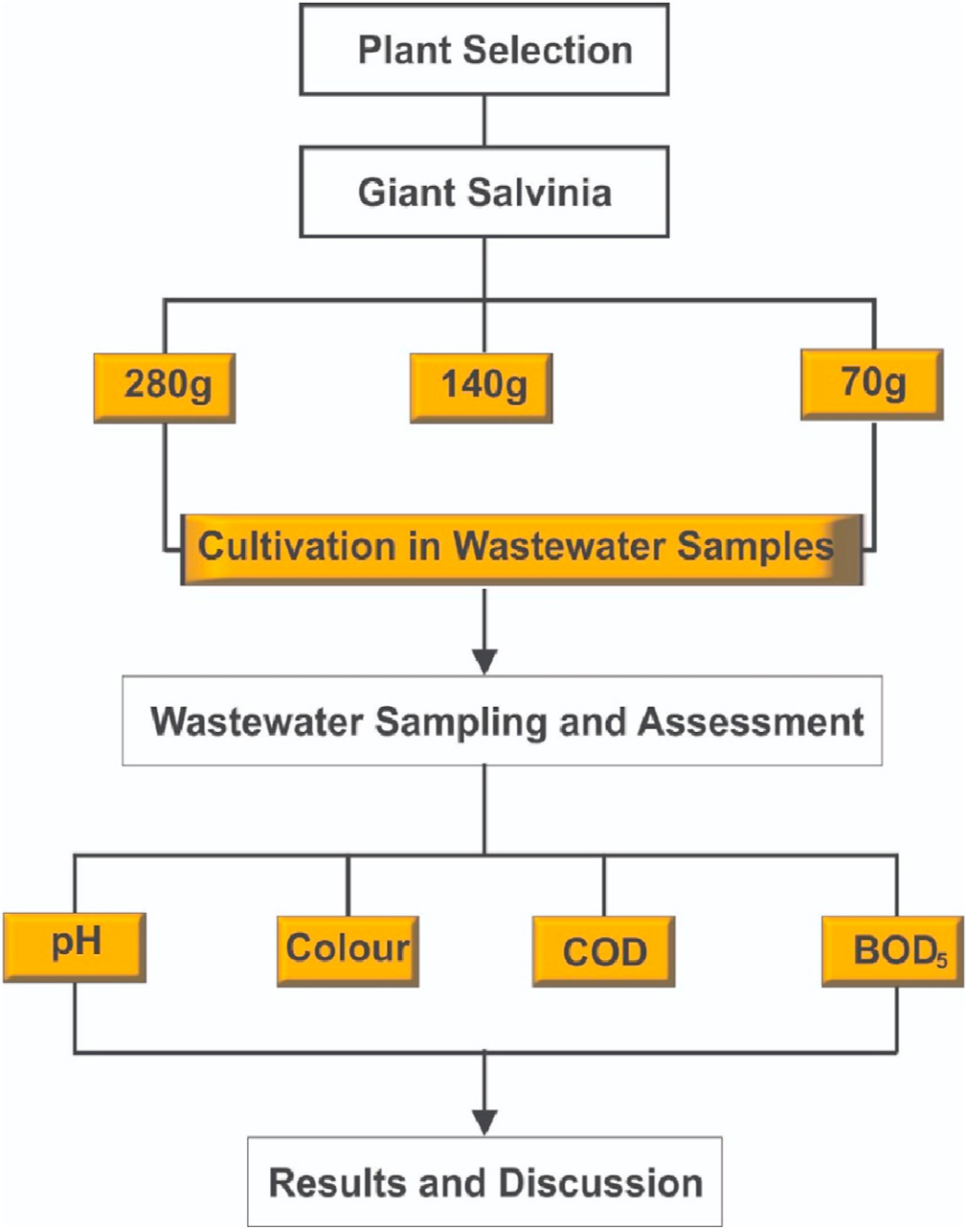
Flow chart of experimental process.

The percentage removal efficiency was calculated according to equation (Eq. 2) [19]. Furthermore, one-way analysis of variance (ANOVA) (IBM SPSS version 25 package, New York, NY, USA) and t-Test at 95% confidence level was applied in obtaining the significance of difference between the influent and effluent water samples. Also, both the mean and standard deviations were calculated using Microsoft® Excel statistical package. The flow chart of the experimental process is illustrated in Figure 3.

Eq. 2 $$\text{Percentage removal efficiency }\left(\text{\%}\right)\;=\;\frac{Qi-Qe}{Qi}\times 100$$

Where Qi = influent concentration.

Qe = effluent concentration

## 3 Results and discussion

The results of the physicochemical analysis are presented in the subsequent sections.

### 3.1 pH analysis of the wastewater samples

**Figure 4:**
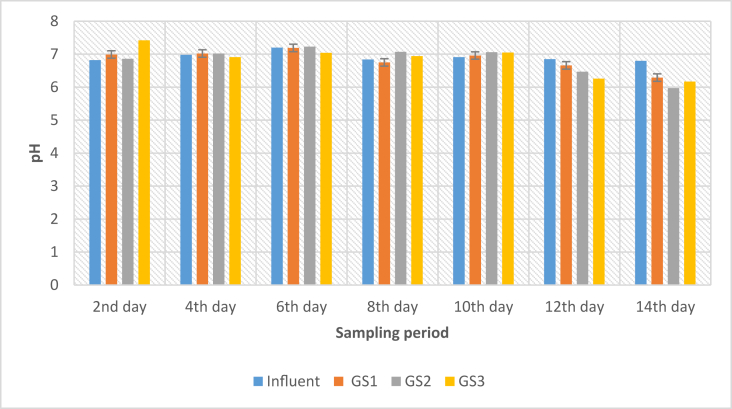
Plot of Graph of pH against Sampling Period.

The performance of *S. molesta* plants in improving the quality of the treated domestic wastewater samples were studied. The outcome of the pH analysis is presented in Figure 4.

From the graph (Figure 4), the results presented indicated insignificant changes in the pH analysis of the influent and effluent water samples. It could be seen that the pH of the influent samples from the STP varied throughout the sampling period. However, the pH of the effluent samples ranged from 5.97 ± 0.02 to 7.42 ± 0.17 during the 14 days treatment process. A slight fluctuation of the pH was observed in the effluent samples when compared with the influent samples. Furthermore, water samples that fall within the pH value of 6–9 is safe to be released into the environment [20]. Also, the slight changes observed in the pH can be attributed to the absorption of contaminants by the *S. molesta* plants. Moreover, the outcomes of the present study agree with the reports of [21,22]. Additionally, the pH of water is a critical factor that determines the biochemical processes of water because the rate of pH influences the rapid breakdown of organic matter and nutrients [23]. Similarly, the decrease in pH will enhance the decomposition of the BOD and COD present in wastewater by microorganisms [21,22]. Furthermore, Safauldeen et al. [24] evaluated the efficiency of water hyacinth in the remediation of batik sewage. The results obtained indicated that pH of 6.8–8.1 was observed after 28 days of exposure. The authors concluded that the maximum efficiency of colour and COD removal in the batik sewage were achieved on the 7^th^ day with 83% and 89%, respectively.

### 3.2 Colour concentration analysis

**Figure 5:**
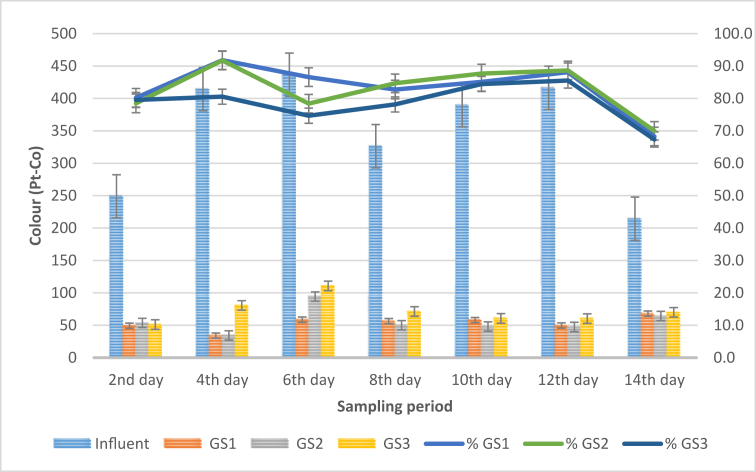
Graph of colour concentration against sampling period.

Colour analysis is an important parameter in determining wastewater quality. Colour concentration in wastewater is associated with the amount of suspended and dissolved solids in water. Figure 5 represents the outcome of the colour analysis conducted on the influent and effluent water samples.

From Figure 5, the trends of the colour reduction demonstrated by GS1 is slightly higher than that of GS2 and GS3 treatment systems. The graph demonstrated that the average colour concentration of the influent samples from the STP varied throughout the sampling period and ranged between 214.3 ± 0.57 to 436.6 ± 1.52 Pt–Co. However, the influent samples have the highest colour concentrations throughout the experimental days. Also, it was observed that GS1 treated samples recorded colour reduction of 49.3 ± 2.08 Pt–Co, 51 ± 3 Pt–Co for GS2 and 53.6 ± 1.52 Pt–Co for GS3 effluent samples as against the influent colour value of 249 ± 2.64 Pt–Co on the 2^nd^ day of the sampling period. Similarly, on the 14^th^ day of the sampling period, *S. molesta* plants lowered the colour concentration of the influent samples from 214.3 ± 0.57 Pt–Co to 68 ± 3, 64.3 ± 1.15 and 70 ± 2.64 Pt–Co for GS1, GS2 and GS3 respectively. The maximum removal efficiency was obtained in GS1 (91.8%) and GS2 (91.7%) treatment systems on the 4^th^ day of the sampling period. However, the performance of the influent and the individual test plants was found to be significant (p < 0.05). Furthermore, the overall average colour value for GS1, GS2 and GS3 was found to be 53.4 Pt–Co, 56 Pt–Co, and 72 Pt–Co respectively as against the average value of 349.4 Pt–Co of the influent samples. Similarly, the variations in the weight of the *S. molesta* plants greatly affected the reduction of the colour concentration, as the treatment system with the lowest density of plants (GS3) recorded its maximum reduction efficiency of 85.5% on the 12^th^ day of the sampling period. In addition, the findings obtained in this study corroborated with the work of [11], who stated that phytoremediation techniques depend on the weight of the plants and contact time. Finally, based on these findings the colour concentration of the influent samples was improved by the test plants and the optimum reduction of colour concentration can be achieved within 4 days at 24 h retention time.

### 3.3 Chemical oxygen demand (COD) analysis

**Figure 6:**
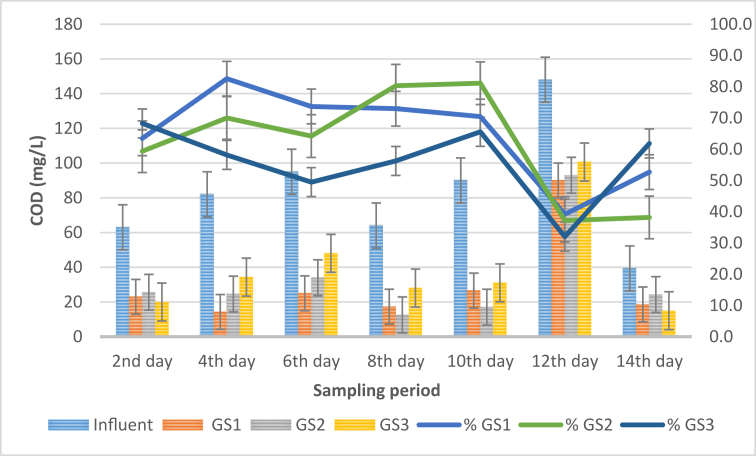
COD concentration against sampling period.

Chemical oxygen demand is a parameter that indicates pollution levels in wastewater samples based on chemical characteristics [25]. The average COD levels of the influent and effluent water samples is represented in Figure 6.

From Figure 6, it is evident from the graph that *S. molesta* plants reduced the COD concentration of the influent samples from the first day onward. The average COD concentration of the influent samples obtained from the STP varied throughout the 14 days sampling period. However, the COD level of the influent sample was reduced from 63 ± 0 mg/L to 23 ± 0 mg/L, 25.6 ± 0.57 mg/L, and 20 ± 0 mg/L by GS1, GS2 and GS3 plants, respectively. Similarly, steady decrease was observed in the GS1 and GS2 treatment systems, but on the 10^th^ day, a drastic decline in the COD level was observed in the two systems. Likewise, fluctuations in the rate of COD reduction was recorded in the GS3 treatment systems. The maximum COD reduction efficiency of 82.6% was observed in GS1 treatment systems on the 4^th^ day of the sampling period. At the same time, the minimum COD reduction was found to be 32% in the GS3 treatment systems. However, the performance between the influent and the individual test plants was found to be significant (p < 0.05). Also, the ANOVA tests between GS1 and GS3 was found to be significant, but an insignificant change (p > 0.05) was observed between GS1vs GS2, and GS2 vs GS3. Furthermore, the average COD value for GS1, GS2 and GS3 was found to be 30.6 mg/L, 33 mg/L and 40 mg/L, respectively as against the overall average COD value (83 mg/L) of the influent water samples. Thus, it is evident from these findings that the varied weight of the plant samples is a factor that determines the performance and the rate at which the plants lowered the COD concentrations of the water samples. Therefore, the outcome of this present study indicated that GS1 treated water samples is within Class II (10–25 mg/L) of the water quality standard for Malaysia. While, GS2 and GS3 treated samples fall within Class III (25–50) and Class IV (50–100 mg/L) category of the Malaysia water quality standards [20]. Similarly, the high rate of COD reduction observed in GS1 and GS2 treatment systems can be attributed to the density of the root system. Gopal [26], reported that the roots of aquatic plants provide a conducive ecosystem for the decomposition of excess nutrients by aerobic microorganisms into organic compounds. These organic matters act as food for the plants. Additionally, the results obtained in this research is in agreement with the work of [27], who reported that COD reduction varied from 49.02% to 98.96% in the treatment of household wastewater using artificial wetland system. Also, Valipour et al. [28] studied the performance of *E. crassipes* in phytoremediation of domestic sewage. The results obtained indicated up to 81% and 91% removal of COD and BOD, respectively.

### 3.4 Biochemical oxygen demand (BOD_5_) analysis

**Figure 7:**
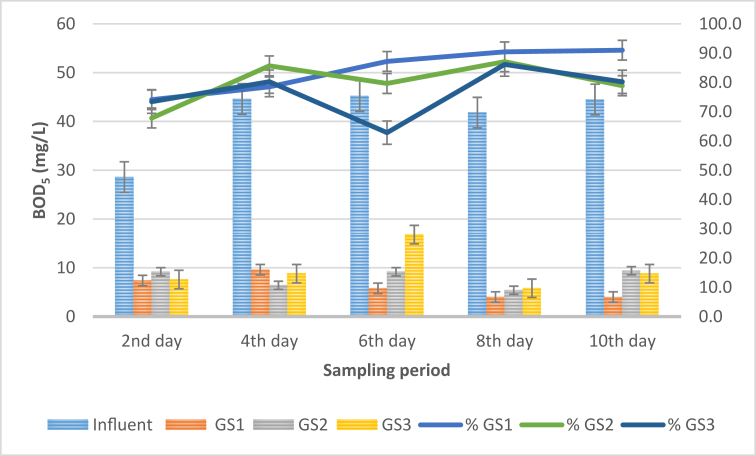
Graph of BOD_5_ concentrations against sampling period.

The present study evaluated the efficiency of different weight of *S. molesta* plants in improving the BOD concentration of the influent and effluent water samples at a retention time of 24 h. Figure 7 represents the outcome of the BOD_5_ analysis.

According to the results presented in Figure 7, the introduction of *S. molesta* plants into the influent water samples has remarkably improved the BOD concentration of the effluent water samples. The trend of the graph showed that the BOD_5_ concentration of the influent samples collected from the STP varied throughout the sampling period. On the 2^nd^ day of the sampling, the test plant decreased the BOD of the influent samples from 28.6 mg/L to 7.4 mg/L (GS1), 9.2 mg/L (GS2), and 7.6 mg/L (GS3). Similarly, on the 8^th^ day of the sampling period, the BOD_5_ of the influent samples was lowered from 41.8 ± 0 mg/L to 4 ± 0 mg/L (GS1), 9.4 ± 0 mg/L (GS2) and 8.8 ± 0 mg/L (GS3). However, the trend of the percentage efficiency indicated that GS1 exhibits the highest reduction efficiency with 91%, followed by GS2 (87%) and GS3 (86.1%). Additionally, the performance of the test plant (effluent samples) indicated a significant change (p < 0.05) when compared to the influent water samples. Furthermore, the average BOD_5_ value was found to be 6 mg/L (GS1), 8 mg/L (GS2) and 9.56 mg/L (GS3) as against the overall average BOD_5_ value (41 mg/L) of the influent water samples. The introduction of the test plants in the treated secondary domestic wastewater has shown up to 91%, 87% and 86.1% reduction efficiency for GS1, GS2, and GS3 treatment systems, respectively. Therefore, among the three treatment systems, GS1 has shown better and steady trends in the reduction of the BOD_5_ level at 24 h retention time than GS2 and GS3 treatment systems. Hence, the optimum conditions for BOD_5_ reduction can be achieved in the GS1 treatment systems. Besides, the effluent water quality standard obtained from the present study falls within the Class III and IV category for BOD water quality standards [20]. In addition, high BOD indicate that sufficient quantity of oxygen is required to decompose the high organic material present in the wastewater. Furthermore, Yasar et al. [29] recorded up to 82% BOD removal in phytoremediation of wastewater using constructed wetlands systems. Moreover, the remarkable reduction of COD and BOD_5_ concentrations observed in the effluent samples might be attributed to the availability of sufficient amount of oxygen and surface area provided by the *S. molesta* plants which enhanced the microbial activities.

## 4 Conclusion

This study investigated the potentials of different weight of *S. molesta* plants in biological treatment of domestic wastewater. The outcome of this study has demonstrated that the quantity of plant determines its performance in nutrient uptake during phytoremediation processes. In other words, GS1 removed 96.8% (colour), 91% (BOD_5_), and 82.6% (COD). While up to 88.6% (colour), 87.1% (BOD_5_), and 81.1% (COD) reduction was observed for GS2 treatment systems, and GS3 was efficient in removing 85.5% (colour), 86.1% (BOD_5_), and 68.3% (COD). Also, a pH value of 6.29–7.19, 5.97–7.07, and 6.17–7.42 was obtained from GS1, GS2 and GS3 treatment systems, respectively. Therefore, the treatment system with the highest quantity of *S. molesta* (GS1) demonstrated better performance compared to the other two systems (GS2 and GS3). Similarly, this study is beneficial to the society as it provides a cost-effective, energy-efficient and environmentally friendly method for tertiary treatment and recycling of wastewater. Also, the harvested *S. molesta* plants obtained after the phytoremediation process can be used as biomass for biofuel generation. Additionally, this research addresses the goals of sustainable development through the use of green technology to reduce the threat of water pollution in natural water bodies.

## Declarations

### Author contribution statement

H.M. Mustafa: Performed the experiments; Analyzed and interpreted the data.

G. Hayder: Conceived and designed the experiments; Contributed reagents, materials, analysis tools or data; Wrote the paper.

### Funding statement

This work was supported by BOLD RESEARCH GRANT 2020 from 10.13039/501100008561Universiti Tenaga Nasional (UNITEN).

### Data availability statement

The authors do not have permission to share data.

### Declaration of interests statement

The authors declare no conflict of interest.

### Additional information

No additional information is available for this paper.
